# Sound Sleep

**Published:** 1856-12

**Authors:** 


### SOUND SLEEP.

Any man who can bound out of bed as soon as he wakes of a mid-winter's morning is worth something; no fear of his not making his way through the world creditably, because he has the elements of a promptitude, decision and energy, which guarantee success. To invalids we make a comfortable suggestion worth knowing. If you have force of will enough to keep you from taking a second nap—and it is the "*second nap*" which makes its baneful influence felt on multitudes—it is better for you to lie awhile and think about it, until that feeling of weariness passes out of the limbs which you so commonly feel. But to sleep soundly, and to feel rested and refreshed when you wake up of a morning, four things are essential—

1. Go to bed with feet thoroughly dry and warm.

2. Take nothing for supper but some cold bread and butter and a single cup of weak warm tea of any kind.

3. Avoid over fatigue of body.

4. For the hour preceding bedtime, dismiss every engrossing subject from the mind, and let it be employed about something soothing and enlivening in cheerful thankfulness.

